# First on-line detection of radioactive fission isotopes produced by laser-accelerated protons

**DOI:** 10.1038/s41598-020-74045-5

**Published:** 2020-10-14

**Authors:** Pascal Boller, Alex Zylstra, Paul Neumayer, Lee Bernstein, Christian Brabetz, John Despotopulos, Jan Glorius, Johannes Hellmund, Eugene A. Henry, Johannes Hornung, Justin Jeet, Jadambaa Khuyagbaatar, Lotte Lens, Simon Roeder, Thomas Stoehlker, Alexander Yakushev, Yuri A. Litvinov, Dawn Shaughnessy, Vincent Bagnoud, Thomas Kuehl, Dieter H. G. Schneider

**Affiliations:** 1 GSI Helmholtzzentrum für Schwerionenforschung, Planckstraße 1, 64291 Darmstadt, Germany; 2 Technische Universität Darmstadt, Institut für Kernphysik, Schloßgartenstraße 9, 64289 Darmstadt, Germany; 3 Lawrence Livermore National Laboratory, 7000 East Avenue, Livermore, CA 94551 USA; 4 Goethe-Universität Frankfurt, 60323 Frankfurt am Main, Germany; 5 Helmholtz Institute Jena, Fröbelstieg 3, 07743 Jena, Germany; 6 Friedrich-Schiller-Universität Jena, Fürstengraben 1, 07743 Jena, Germany; 7 Johannes Gutenberg-Universität Mainz, Saarstraße 21, 55122 Mainz, Germany

**Keywords:** Plasma-based accelerators, Nuclear physics, Nuclear astrophysics

## Abstract

The on-going developments in laser acceleration of protons and light ions, as well as the production of strong bursts of neutrons and multi-$\hbox {MeV}$ photons by secondary processes now provide a basis for novel high-flux nuclear physics experiments. While the maximum energy of protons resulting from Target Normal Sheath Acceleration is presently still limited to around $100 \, \hbox {MeV}$, the generated proton peak flux within the short laser-accelerated bunches can already today exceed the values achievable at the most advanced conventional accelerators by orders of magnitude. This paper consists of two parts covering the scientific motivation and relevance of such experiments and a first proof-of-principle demonstration. In the presented experiment pulses of $200 \, \hbox {J}$ at $\approx \, 500 \, \hbox {fs}$ duration from the PHELIX laser produced more than $10^{12}$ protons with energies above $15 \, \hbox {MeV}$ in a bunch of sub-nanosecond duration. They were used to induce fission in foil targets made of natural uranium. To make use of the nonpareil flux, these targets have to be very close to the laser acceleration source, since the particle density within the bunch is strongly affected by Coulomb explosion and the velocity differences between ions of different energy. The main challenge for nuclear detection with high-purity germanium detectors is given by the strong electromagnetic pulse caused by the laser-matter interaction close to the laser acceleration source. This was mitigated by utilizing fast transport of the fission products by a gas flow to a carbon filter, where the $\upgamma$-rays were registered. The identified nuclides include those that have half-lives down to $39 \, \hbox {s}$. These results demonstrate the capability to produce, extract, and detect short-lived reaction products under the demanding experimental condition imposed by the high-power laser interaction. The approach promotes research towards relevant nuclear astrophysical studies at conditions currently only accessible at nuclear high energy density laser facilities.

## Introduction

At present, Target Normal Sheath Acceleration (TNSA)^1, 2^ produces protons with energies up to around $100 \, \hbox {MeV}$^3^, with abundances of more than $10^{12}$ protons above $15 \, \hbox {MeV}$^4^. One of the pre-requisites for TNSA is the necessity to maintain relativistic intensities during the laser-matter interaction over many tens of femtoseconds, a capability which is met by low-repetition-rate high-intensity lasers of $10 \, \hbox {Hz}$ and below. This acceleration scheme opens up new opportunities for proton-induced reactions that until now have not been possible. Indeed, while the average proton flux of a laser-based proton source remains low compared to standard accelerators, the peak proton flux reaches $10^{22}$ particles per second within the laser-accelerated particle bunch, which dramatically exceeds the capabilities of conventional particle accelerators. This gives a perspective to probe into new regimes or reliably trigger and detect processes with small cross-sections at ultrashort lifetimes. Additional improvements of these parameters by novel laser-driven acceleration schemes, although not yet substantiated, are expected and will increase the proton flux^5, 6^. A requirement is the combination of the laser production method with spectroscopy setups and tools, allowing for sensitive nuclear spectroscopy on nuclides with a short half-life. In order to explore this new field, a platform had to be developed that can detect $\upgamma$-ray and $\upbeta$-particle emission from the fission fragments in the presence of the strong electromagnetic pulse (EMP) created by the laser-matter interaction. Such a platform based on the radiochemistry method opens up the possibility for the investigation of nuclear excitation processes at the nuclear-atomic interface in high-energy-density (HED) environments^7^.

This present experiment, conducted at the PHELIX facility^8^, the Petawatt High-Energy Laser for Heavy-Ion eXperiments at GSI in Darmstadt, Germany, is part of the early development phase of a platform for future nuclear physics experiments at other advanced laser facilities (e. g. ELI-NP (Romania)^9, 10^ and ARC (LLNL)^11^). Due to its continuous gas flow, the method used is also well suited for high repetitive lasers like ELI-Beamlines (Czech Republic)^12^, BELLA (LBNL)^13^, Draco (HZDR)^14^, J-KAREN (KPSI)^15^, and CoReLS (South Korea)^16^. The platform utilizes a state-of-the-art short pulse, high temporal contrast, high energy laser (in the experiment $\approx \,200 \, \hbox {J}$ in $\approx \, 500 \, \hbox {fs}$ at $<\, 10^{-12}$ temporal contrast) to provide laser-accelerated protons to induce nuclear reactions. The rapid high-efficiency detection of reaction products is the primary focus.

In this experiment, we studied proton-induced fission of uranium, motivated by the appropriate matching of the wide cross-section profile to the range of the proton spectrum produced by TNSA. A particularly interesting aspect of these experiments with short-pulsed, laser-driven particle beams is that the pulse duration is much shorter than in conventional sources and limited only by the laser pulse itself and the associated time-of-flight spread of the accelerated ions. An abundance of fission fragments is produced within this time, providing sensitivity to short-lived isotopes. Nuclei with half-lives down to several seconds have been identified. So far, very little experience exists in this short-pulsed time domain. The transportation of the fragments to the detection apparatus can take several seconds, which ultimately impairs sensitivity to very short-lived isomers. Nevertheless, the build-up of daughter isotopes from the chain-yield and the $\upgamma$-rays resulting from their deexcitations can be expected in the measured spectra, potentially revealing heretofore unobserved aspects of the reaction process. Our successful proof-of-principle experiment opens a new perspective for a variety of nuclear structure and nuclear astrophysics, where some of the very small reaction cross-sections with shortest lifetimes require huge luminosities not yet available at conventional accelerators.

## Results and discussion

The purpose of this work is to demonstrate the use of laser-accelerated high-flux proton bursts for nuclear physics applications. The immediate goal of this first beam time was to observe short-lived ${}^{\mathrm{nat}}$U(p,f) fission products. Nuclides with half-lives as short as $39 \, \hbox {s}$ were readily identified in the measured spectra. Observation of products with even shorter half-lives requires an upgraded detector setup planned in future studies.

**Figure 1:**
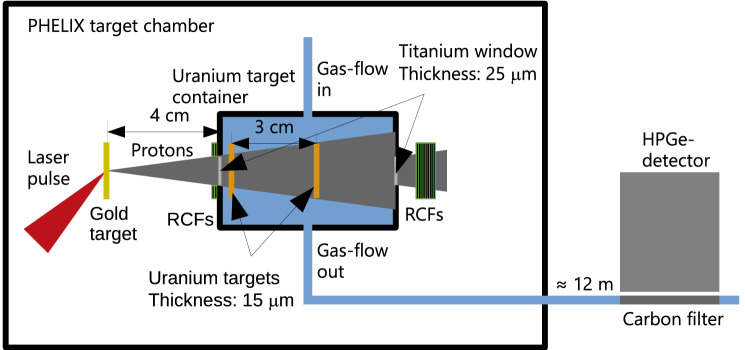
Experimental setup. The produced protons from TNSA enter the uranium-target container through a titanium window. RCFs located in front and behind the uranium-target container measure the proton spectrum. The fission products are stopped by a gas mixture of helium and argon in the same mass flow proportion and transported to a carbon filter $12 \, \hbox {m}$ away outside the PHELIX target chamber. The various $\upgamma$-lines from the transported isotopes are measured with a HPGe detector.

**Figure 2:**
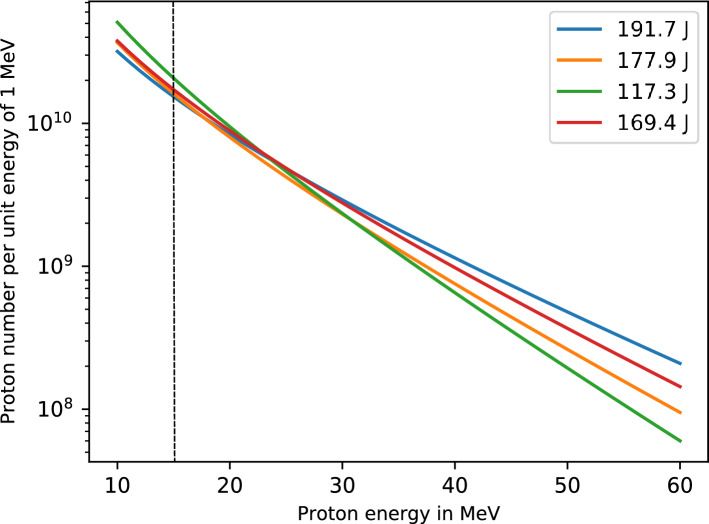
Proton spectrum of calibration laser shots recorded with RCFs $5 \, \hbox {cm}$ behind the laser acceleration source. To reflect the aperture of the uranium target container, only $1 \, \hbox {cm}$ of the RCFs in the middle were evaluated. The exponential function is obtained by an iterative t to the deposited energy in the RCF layers. Above the dotted line at $15 \, \hbox {MeV}$ the protons contribute significantly to the fission process.

Using the setup described in Fig. 1, two 15-$\upmu$m-thick uranium foils located 50 and $80 \, \hbox {mm}$ away from the proton source were irradiated by $(1.35 \pm 0.12)\, \times \, 10^{11}$ and $(0.506 \pm 0.047)\, \times \, 10^{11}$ protons with energies above $15 \, \hbox {MeV}$, respectively, taking into account the solid angle of irradiation. Figure 2 gives the retrieved proton spectra for calibration shots using radiochromic films at imaging spectroscopy (RIS) at the distance of the first uranium foil. The exponential function is obtained by an iterative fit to the deposited energy in the RCF layers. Altogether, this illustrates the statistical and systematic sources of error made in estimating the proton numbers. Applied to the uranium target, this corresponds to an effective conversion efficiency of $(0.50\pm 0.16) \%$ at the first uranium foil and of $(0.19 \pm 0.06) \%$ at the second, and represents the fraction of laser energy that is converted into usable kinetic energy. The cross-section of proton-induced fission reaches $500 \, \hbox {mb}$ around $15 \, \hbox {MeV}$ and is rising to a maximum of $1.75 \, \hbox {b}$ at $70 \, \hbox {MeV}$^17^, well matching the energy spectrum achieved. The most energetic protons arrive at the first uranium foil in $0.46 \, \hbox {ns}$ subsequent to laser irradiation of the gold target, while less energetic protons with $E= 15 \, \hbox {MeV}$ arrive at $0.94 \, \hbox {ns}$. The overall exposure of uranium to the proton flux was shorter than $1 \, \hbox {ns}$, which corresponds to an effective peak current in excess of $10^{20} \, \hbox {protons/s}$. During the irradiation, gas is constantly flushed through the uranium target container resulting in the transport of the freshly produced fission fragments to a carbon filter located directly in front of an HPGe detector.

**Figure 3:**
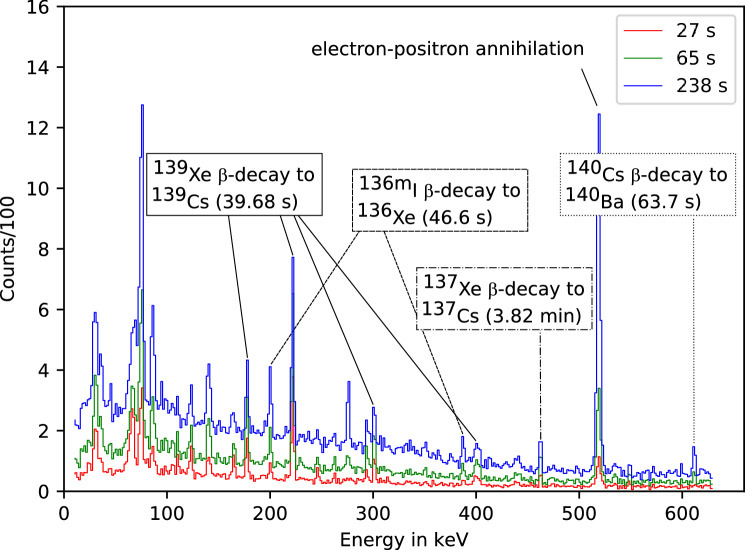
Measured $\upgamma$ spectra and some identified fission products. The spectra were accumulated for periods of 27 (red), 65 (green), and 238 (blue) seconds following the laser pulse. For these spectra, the results of eleven laser shots are added up.

The $\upgamma$ spectra recorded by the HPGe detector were analyzed on the basis of published nuclear data tables^18, 19^ for the identification of the observed $\upgamma$-ray peaks, and associated lifetimes. All major volatile fission product isotopes having a lifetime in the range of $39.68 \, \hbox {s}$ to $14.14 \, \hbox {min}$ were identified. These are: ${}^{134\mathrm{m}}$I, ${}^{136\mathrm{m}}$I, ${}^{137}$Xe, ${}^{138}$Xe, ${}^{139}$Xe, and ${}^{140}$Cs. Figure 3 depicts the $\upgamma$-ray spectra recorded at three different times, 27 (red), 65 (green), and 238 (blue) seconds, respectively, following the laser pulse. The time information facilitates the identification. Each spectrum represents an accumulation of detected $\upgamma$-rays up to the indicated times. The background in the spectra is low, due to the short integration time and the ability to massively shield the detector against the EMP and high-energy radiation. Due to a large number of various radioactive nuclei produced in this experiment, we focused on unambiguous identification of abundantly generated fission products and did not aim at identification of all $\upgamma$-lines in the spectra. Broader features at low energies may stem from X-ray emission following internal conversion and positron decays, and secondary excitation from the lead shielding. Neutron-deficient nuclides responsible for the latter decays are probably produced in proton-induced direct reactions like (p,pn), (p,2n) or (p,3n). Also, the fragmentation or spallation reactions due to incident protons are not excluded. The presence of such neutron-deficient nuclides is evidenced by a relatively strong $\upgamma \hbox {-line}\, =\, 511 \, \hbox {keV}$ responsible for positron-electron annihilation. For these spectra, the results of eleven laser shots are added up. It should be noted that the $\upgamma$-lines associated with the nuclei ${}^{134\mathrm{m}}$I, ${}^{136\mathrm{m}}$I, and ${}^{139}$Xe can be clearly extracted even from a single shot, as they are produced with high enough yield.

Table 1 lists the identified $\upgamma$-rays present in the spectra. Given are the parent isotope, the respective decay mode, the associated lifetime, and the $\upgamma$ energy as well as the number of collected nuclides in the filter and the integral cross-sections. Other $\upgamma$-ray energies are expected from isotopes produced later in the decay chain but are not visible due to the selected time window.

**Table 1:** Identified nuclides with their lifetimes, their excited daughters and their $\upgamma$-ray energies from literature^18, 19^ as well as the number of collected nuclides per shot in the filter and the calculated integral cross-sections.

Parent decay	Lifetime	Excited daughter	Gamma in keV	Number of collected nuclides per shot	Integral cross-section in mb
${}^{134\mathrm{m}}$I	$3.52 \, \hbox {min}$	${}^{134}$I	272.1	551 ± 59	1.17 ± 0.10
${}^{136\mathrm{m}}$I	$46.6 \, \hbox {s}$	${}^{136}$Xe	197.32, 381.36, 1313.02	147 ± 16	0.636 ± 0.051
${}^{137}$Xe	$3.82 \, \hbox {min}$	${}^{137}$Cs	455.49	860 ± 130	2.04 ± 0.24
${}^{138}$Xe	$14.08 \, \hbox {min}$	${}^{138}$Cs	434.56	750 ± 400	1.09 ± 0.38
${}^{139}$Xe	$39.68 \, \hbox {s}$	${}^{139}$Cs	174.97, 218.59, 289.78 296.53, 393.5	627 ± 47	3.13 ± 0.17
${}^{140}$Cs	$63.7 \, \hbox {s}$	${}^{140}$Ba	602.35	529 ± 75	1.85 ± 0.20

**Figure 4:**
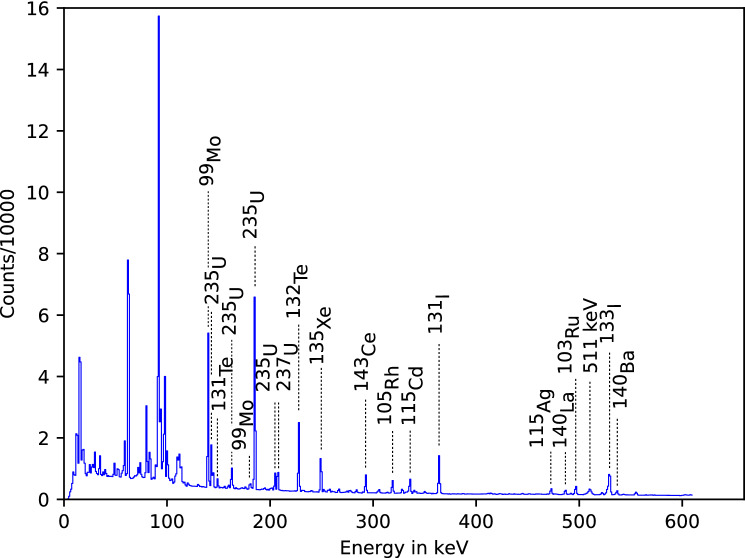
Measured $\upgamma$ spectrum of the irradiated uranium targets after the experiment after an accumulation period of $81 \, \hbox {min}$.

For comparison, Fig. 4 shows the spectrum of the irradiated uranium targets after the experiment. In this spectrum, a large number of reaction products can be identified, including long-lived daughter nuclei from the decay chains of fission fragments and nuclides generated by a direct reaction like ${}^{237}$U from ${}^{238}$U(p,pn) and ${}^{236}$Np from ${}^{238}$U(p,3n). In addition, $\upgamma$-lines from the natural decay of uranium appear. Since the spectrum was taken several hours after the irradiation, short-lived isotopes are not visible. This demonstrates and validates that the experimental setup enables the detection of short-lived isotopes, that can otherwise not be identified by post-irradiation measurements.

One interesting feature of this experimental platform is the possibility to calibrate the various modules off-line and deliver quantitative reaction yield values. The proton source is calibrated with radiochromic films (RCFs), which is an established calibration method. RCFs are readily available for medical applications from various vendors. By calibrating the RCFs against an external accelerator source, an absolute error in dose deposition around $5 \%$ is normally achieved. At the other end of the system, the detector efficiency was cross-calibrated using radioactive sources. Additionally, the losses in the gas transport in the capillary were determined off-line for ${}^{219}$Rn to be less than $1 \%$ including the capture efficiency in the carbon filter. Given that the container reaction geometry is well known, this platform could be used to determine integral reaction cross-sections quantitatively. For this purpose, the stopping range of the generated fission fragments and the proportion of the generated fission fragments that can enter the gas was determined. The number of target particles available for the measured fission products is $(1.71\pm 0.11)\,\times \, 10^{19}$ for iodine and $(1.56\pm 0.11)\, \times \, 10^{19}$ for xenon depending on the different stopping range. $10.27 \%$ of the iodine and $11.22 \%$ of the xenon elements diffuse into the gas and have the possibility to be stopped there. It should be noted, that the error of the cross-sections are mainly statistical, which can be reduced by a longer series of measurements and by adding up several spectra of laser shots. The ratios between the isotopes are completely consistent with literature values^20, 21^. The measured cross-sections are too low by a factor of 8. This has to be explained by deficiency of the extraction from the target container.

It is important to emphasize that the ability to identify shorter-lived isotopes is improved when lasers such as PHELIX are utilized. In our realization, the lower limit of observed lifetimes and the restriction to transport only volatile elements were dictated by the specific conditions chosen for the gas transport parameters, aiming for improved operation safety. By changing these parameters, much faster transport times can be achieved.

## Scientific motivation and relevance

Laser-driven ion acceleration produces particle bunches of unprecedented density. In a secondary target that is close enough to the laser acceleration source, this leads to a nuclear-interaction-rich scenario. However, this environment is not background free and hinders a direct detection of the processes of interest. In combination with a gas transport system, we achieved to transfer the reaction products to a safe location for typical HPGe detection, more than $10 \, \hbox {m}$ away from the point of laser interaction. This allowed for high-detection sensitivity and nearly background-free $\upgamma$ spectroscopy while mitigating the interference of the strong EMP.

High-flux proton pulses of sub-nanosecond down to picosecond duration provide an opportunity to study nuclear reactions in a plasma-like environment. For example, this pulsed-beam regime could cause additional Coulomb excitations of the target material by the impinging protons within the pulse duration, similar to the low-lying states’ coupling effects in highly-charged states heavy-ion reactions. Effects on the cross-sections from varying excited state populations due to the plasma environment will be of interest in future experiments.

### Fission in HED environment

For fissionable nuclei embedded in a HED environment, the plasma interaction is expected to affect “transition” states and alter the fission probability. The initial studies involve measurements of the isotope distributions of fission fragments identified from the observation of characteristic $\upgamma$-rays. In the nuclear fission process, a heavy nucleus typically separates into energetic fission fragments with different masses sharing a total kinetic energy gain of approximately $200 \, \hbox {MeV}$^22, 23^. Details of the induced fission process over a wide range of projectile species and energies are still not well understood for many fissionable nuclei. Furthermore, a fully microscopic theory of fission describing experimental data is still to be developed. Accurate measurements of cross-sections as well as new techniques to measure short-lived fission fragments will provide new insight into the nucleon-nucleus interaction. In particular, information on the properties and effects of highly excited nuclei, e.g., level densities and fission barriers, can be gained.

Neutron-induced fission experiments at the National Ignition Facility (NIF) have demonstrated the capability to rapidly collect and detect short-lived gaseous fission fragment isotopes for the first time, following an approximately 80 ps-long pulse of nearly $10^{16} \, 14{\rm {-MeV}}$ neutrons into $4\pi$ from a burning deuterium-tritium fusion plasma^23^. The neutron-induced fission at NIF, and the present experiment at PHELIX, demonstrate fission studies in a new time-domain amid plasma environments.

Fission research still is intensively pursued. A particular interest involving astrophysical HED environments now is driven by energy research, e.g., novel nuclear reactor schemes^24^, advanced nuclear astrophysics, and cosmogenic nucleosynthesis (e.g., r-processes)^25^. The long history of fission research and the renewed interest reveal that there are many open and new questions regarding fission in HED environments. The detection of plasma effects on nuclear reactions, for example, its influence on the nuclear fission process’s mechanisms, is a long-term focus at HED facilities. Since the discovery of fission in the 1930s, extensive data and literature on fission have been published^19, 22, 26^. The nuclear fission is treated within the macroscopic-microscopic nuclear models. Here, the macroscopic part is a charged liquid drop described by the standard formula for nuclear ground state energies: $E = E_\text {Vol} + E_\text {Surf} + E_\text {Coul} + E_\text {Pauli}$. The terms correspond to effects arising from the nuclear volume, the surface area, Coulomb repulsion, and the Pauli exclusion principle (asymmetry energy), respectively. The microscopic corrections, shell effects (utilizing the Nilsson model), and the pairing interaction, are taken into account^22^ as well as, most recently, fragment formation^27^. This led to a satisfactory description of the fission barrier with an appropriate height as well as oscillation (“hump structure”) of the potential energy barrier for actinide nuclei as a function of nucleus deformation^26^.

Motivated through new nuclear astrophysical observations, recent research on macroscopic-microscopic nuclear models aimed to improve the understanding of fission, the fission barriers, and dynamic aspects of fission in HED environments^25^. The heavy element nucleosynthesis in neutron star mergers involving fission and fission recycling are examples. For “multiple-hump” actinide fission barrier structures, the second potential minimum has been extensively investigated in fission isomer studies. The existence of a third potential minimum has recently been established for even-even uranium nuclei (as well as an odd isotope). The thin outer barriers result in shorter fission lifetimes than those from collectively excited states^25^. The detailed studies involve transmission resonance spectroscopy, where the prompt fission cross-section is measured. The cross-section exhibits resonance enhancements at certain excitation energies because the energies of the collective states in the first well coincide with those of the vibrational states in the second or third well. The verification of a third well led to an agreement between measured cross-sections and model calculations with appropriate assumptions for the multiple-hump structure and well depths (e.g. ${}^{232}$Thorium).

### Excited states and nuclear decay

The fission platform being developed will also enable research on the effects of a HED environment on other nuclear processes. In a plasma environment, atomic binding energies are modified due to charge state and screening effects, and atomic transitions may interact with excited nuclear states, thereby causing nuclear transitions^7, 28, 29^. Therefore, electron-mediated NPIs may cause significant changes in reaction cross-sections in HED environments such as astrophysical plasmas. However, NPIs remain largely unobserved due to the extremely narrow energies of nuclear transitions ($\Gamma \le 1\, \upmu \hbox {eV}$). Various attempts to detect NPIs such as NEEC (Nuclear Excitation by Electron Capture)^29, 30^ or NEET (Nuclear Excitation Electron Transition)^31–34^ processes are in progress but no consistent experimental evidence has been reported yet. In hot stellar environments, the nuclei can reach a thermal population of low-lying nuclear states from photoexcitation^35, 36^, NEEC, NEET, and inelastic electron scattering in the dense stellar plasma. Since the nuclear reaction cross-sections dramatically depend on the spin-parities of the involved quantum states and the reaction Q-value, studies of nuclear reactions in hot plasma environments are essential. The relevance of NPI processes to the production of post-capture nuclei is due to a competition between the short lifetimes of highly-excited states versus a large number of nuclear transitions available, and high electron and photon flux.

### Nuclear astrophysics relevance

The nucleosynthesis research on the heavy-element abundance determined by s- and r-processes in supernovae and neutron-star mergers motivates laboratory astrophysics studies. The s-process, in which the time interval between neutron-captures is longer than the average $\upbeta$-decay lifetime, proceeds through nuclides along the valley of stability with an isotopic abundance inversely proportional to neutron-capture rates. The s-process is sensitive to nuclear shell closures at which the neutron capture probabilities abruptly change. The work of the s-process is seen in the measured solar element abundance pattern as sharp peaks at Sr (N = 50), Ba (N = 82) and Pb (N = 126). The faster r-process occurs in high temperature ($> 10^9 \hbox {K}$) and high neutron density ($> 10^{20} \, {\hbox {cm}}^{-3}$) environments in events that last several seconds^37^. A rapid capture succession on a seed nucleus goes until the neutron binding energy is sufficiently small so that the rate of capture is balanced by the photodisintegration by ambient black-body photons. Just as in the s-process, the neutron capture rates significantly change at nuclear closures, leading to so-called “waiting points”. After some time, $\upbeta$-decay occurs, and the capture process starts again. The r-process is terminated by reaching the nuclei that undergo fission, thereby fuelling the r-process by neutrons from fission and moving the abundance pattern to mid-Z nuclei. When the neutron flux ceases, radioactive products decay back to the valley of stability (low-mass side of s-process peaks), and the resulting, broader than in s-process, peaks in the mass distribution are identifiers for the r-process. The path of r-process flow is through neutron-rich nuclei far from the valley of stability through nuclei with binding energies of about 1 to $4 \, \hbox {MeV}$. At this stage, recycling fission may come into play: heavy elements produced by the r-process can undergo fission, enhancing the population of neutron-rich lower-Z elements. The r-process abundances are determined from solar abundances minus s-process contributions. Thus, uncertainties persist in the elemental abundances produced by the r-process.

Substantial experimental and theoretical efforts are proposed and in progress for upcoming new laser facilities (e. g. ELI, ARC) and ion beam facilities (e.g. FAIR) to improve the database and theoretical modeling to understand astrophysical data. Concrete experiments are planned at ELI^10, 38^ to demonstrate the production of neutron-rich nuclei at the N = 126 waiting point with lasers. We believe that the method presented here is ideally suited to detect such products.

In the future, we plan on extending our method to investigate proton-induced reactions of astrophysical importance. Here unprecedented proton intensities might allow us to address reactions with tiny cross-sections that are presently inaccessible at dedicated underground accelerators. For instance, (p,$\upgamma$), (p,n), and (p,$\upalpha$) reactions can be studied simultaneously. However, this might require to perform the laser experiment underground as well. Furthermore, owing to the very short duration of the laser pulse, application of time-of-flight techniques will be pursued to enable energy-resolved measurements.

## Experimental method

A schematic diagram for the experimental setup at the GSI PHELIX facility is depicted in Fig. 1. The setup includes the laser-driven proton source and the uranium target container inside the PHELIX target chamber and the fission isotope collection filter directly in front of the $\upgamma$-detector outside the target chamber.

### Laser-driven proton source and laser setup for TNSA

An extensive work has taken place in the last two decades on the generation of protons with lasers, after the pioneering work done at the Nova-Petawatt laser facility^1^. Since then, the developments have followed two main directions, the first one exploring theoretically and experimentally the various mechanisms underlying laser-driven proton acceleration, with the aim to generate highly-energetic and mono-energetic proton beams in the energy range of 0.1–1 GeV, and the second one is optimizing the proton sources for applications^39^.

As far as laser-driven proton acceleration is concerned, the high-energy sub-picosecond PHELIX system, which is based on a neodymium-doped-glass chain of amplifying modules, has distinct advantages over other short-pulse lasers. First, the laser energy and long interaction times of sub-picosecond lasers enable reaching the highest proton yields as a consequence of the combined effects of the energy and high conversion efficiency demonstrated with this type of laser^40^. Second, some preliminary work has been done at PHELIX, as in the framework of the LIGHT project^41^, to condition laser-generated protons for applications, and this knowledge lays the basis for further work. Previous findings have shown two optimal interaction conditions for the generation of stable and reliable protons beams. The first configuration, used in the LIGHT project, deals with relatively thick (several-micrometers) foils, irradiated with a laser pulse exhibiting a moderate nanosecond pedestal. In this case, the nanosecond pedestal creates a pre-plasma at the surface of the interaction foil that fosters improved light absorption, and therefore a more efficient proton generation. The acceleration takes place in the TNSA regime, and the target thickness helps with the proton beam homogeneity. In the second configuration, the laser irradiates a sub-micrometer or micrometer foil^42^. To avoid target pre-heating that destroys the foil, the level of the pulse pedestal is reduced by the use of an ultrafast optical parametric amplifier in the laser^43^. While the thinner targets deliver higher proton energies, the drawback comes from a lower laser-energy coupling into the target because of the thin nanometer-thick interaction volume at the target surface.

Optimization of the proton source by directly comparing quantitatively these proton-source geometries has been conducted. Our findings show that, for the current PHELIX parameters, a proton source generated by irradiating $1 \, \upmu \hbox {m}$ thick foils with P-polarized laser beams of the highest temporal contrast under $45^{\circ }$ incidence is the best trade-off^44^. After the initial setup and test, the source delivered reliably, on every shot, TNSA proton beams with $10^{12}$ protons of energy higher than $15 \, \hbox {MeV}$ and with cut-off energy around $70 \, \hbox {MeV}$. In comparison to standard experiments, the pulse profile of PHELIX was stabilized using an acousto-optic programmable dispersive filter (Fastlite, France) and the pulse profile (spectrum and phase) adjusted daily directly at the interaction point. A measured proton spectrum is shown in Fig. 2.

### Experimental setup for proton-induced fission

The PHELIX target chamber contains the laser transport and focusing mirrors and diagnostics, as well as the target holder for the proton production. The setup is shown in Fig. 1. A compact target container, holding the uranium target foils, is positioned at a distance of $4 \, \hbox {cm}$ from the laser acceleration source. The proton bunch enters and leaves the uranium target container through 15-$\upmu$m-thick titanium windows. A stack of three radiochromic films (RCFs) is placed in front of the container to obtain a spatial diagnostic of the proton beam. On the backside, a stack of eleven RCFs is mounted, to diagnose the protons after they pass through the uranium targets. The sensitivity of these films was calibrated at a proton accelerator. While the PHELIX target chamber is operated at a vacuum of $10^{-5} \, \hbox {mbar}$, the target container is connected to the gas transport system with a constant gas flow of $500 \, {\hbox {cm}}^{3} \, {\hbox {min}}^{-1}$ at a pressure of approximately 1 bar. The container has a length of $60 \, \hbox {mm}$ and a diameter of $40 \, \hbox {mm}$. Two uranium target foils with a size of $16 \, \hbox {mm}\, \times \, 19 \, \hbox {mm}$ and a thickness of $15 \, \upmu \hbox {m}$ are mounted between two metal frames of size $25 \, \hbox {mm} \, \times \, 20 \, \hbox {mm}$, with a 10-mm opening. Due to their fission energy, the fragments are emitted from a thin layer at the front and back of the target material. To increase the fission fragment production yield, two uranium foils were used with a distance of $30 \, \hbox {mm}$ between them, equivalent to the approximate stopping distance of the fragments. Fission fragments are stopped in the gas and transported through a polytetrafluoroethylene (PTFE) tube with an inner diameter of $4 \, \hbox {mm}$, over a distance of $12 \, \hbox {m}$ to be collected in a carbon filter at room temperature, in front of an HPGe detector system. This method is often practiced for nuclear chemistry experiments^45, 46^ but not adapted so far together with laser acceleration. The transport distance of several meters is crucial in this case in order to reduce EMP-induced background at the HPGe detector to an acceptable level.

In the experiment, helium, nitrogen, neon, and argon were used as a transport gas. Depending on the gas, the stopping power and the background in the spectrum changed, caused by the activation of the carrier gas. The best results were achieved with a mixture of helium and argon with a mass flow ratio of 1:1.

The carbon filter consists of a PTFE tube filled with carbon grains with 20–40 $\upmu \hbox {m}$ mesh held in place on both sides by quartz wool. The capture efficiency for rare heavy gases was tested before the main experiment with ${}^{219}$Rn from an ${}^{227}$Ac source. By comparison of the activity retained in the filter to the activity in a second identical filter, a capture rate of $(99.29\pm 0.15) \%$ was found. For the detection an N-type HPGe detector with electric cooling was used. The recorded spectra were stored at certain time intervals in order to obtain the time-dependent behavior of the spectrum. Even at a distance of $12 \, \hbox {m}$ from the laser target, the detector and its electronics had to be shielded. In addition to a metallic cover, a thin, flexible shielding of polyamide spun-bond fleece, which is internally metal-coated, was used. It offers damping of around $100 \, \hbox {dB}$ over a wide spectral range of electromagnetic radiation. The filter and the detector head were enclosed with lead shielding to minimize the $\upgamma$-ray spectrum background in the relevant energy range. The collected fission products are identified on the basis of the observed $\upgamma$-rays. The most short-lived isotope observed had a lifetime of 39 seconds, due to the transport time.

This transport time is given by the volume of the PTFE tube and the mass flow in the gas system. In the given setup, it is approximately $18 \, \hbox {s}$. A transport time of around one second can be achieved by using a slightly higher pressure in combination with a stronger vacuum pump at the exhaust. The restriction of the present experiment to volatile elements can also be overcome by adding reactive gas or aerosols or a combination of both. For future experiments, it will be possible to use more advanced detection systems^47^, which will allow the detection of electrons and photons and registration of coincidences.
